# Determination of individual factors associated with hallux valgus using SVM-RFE

**DOI:** 10.1186/s12891-023-06303-2

**Published:** 2023-06-29

**Authors:** Hidetoshi Nakao, Masakazu Imaoka, Mitsumasa Hida, Ryota Imai, Misa Nakamura, Kazuyuki Matsumoto, Kenji Kita

**Affiliations:** 1 Faculty of Social Work Studies, Josai International University, Chiba, Japan; 2 School of Rehabilitation, Osaka Kawasaki Rehabilitation University, Osaka, Japan; 3 School of Technology, Industrial and Social Sciences, Tokushima University, Tokushima, Japan

**Keywords:** Feature selection, Hallux Valgus, Manchester Scale, SVM-RFE

## Abstract

**Introduction:**

This cross-sectional study aimed to determine the factors related to hallux valgus (HV) and their importance using support vector machine-recursive feature elimination (SVM-RFE).

**Methods:**

A total of 864 participants aged ≥ 18 years were enrolled. The Manchester scale was used to determine the presence of HV (summed scores for both feet ≥ 4). The questionnaire included items such as age, sex, height, weight, and foot measurements. These internal factors were analyzed to determine if they are related to HV using SVM-RFE.

**Results:**

The results of tenfold cross-validation using SVM-RFE revealed that the numbers of feature selections were 10, 10, and 9 for age, sex, and body weight, respectively, and these factors were shown to be related to HV. HV was found to be more common in women than in men (women, 24.9%; men, 7.6%), but the sex difference was not significant in older people.

**Conclusion:**

Age and sex were found to be important factors associated with HV identified via feature selection using SVM-RFE.

## Introduction

Hallux valgus (HV) is a joint deformity that occurs in the first metatarsophalangeal joint of the great toe, in which the first metatarsal upon birth turns inward and the great toe turns outward. HV is the most common joint deformity, with an estimated incidence of 21–65% [1, 2]. It increases with age and was reported to occur in 23.0% of individuals aged 15–65 years and in 35.7% of those aged ≥ 65 years [2]. Furthermore, it causes foot pain and is correlated with impaired gait and balance, thereby increasing the risk for falls in older people [3]. HV is caused by the displacement of the first ray in the dorsal–medial direction due to the hypermobility of the first tarsometatarsal joint and foot pronation due to loading of their body weights [4, 5].

In general, HV is quantitatively diagnosed based on the dorsal plantar radiographs of the foot during loading. Furthermore, its presence is confirmed if the angle between the long axis of the first metatarsal and the long axis of the great toe is ≥ 15° [6]. However, owing to the difficulty in performing a radiographic examination in epidemiologic studies, self-reports [7], standardized photographs [8], and line drawings are used [9]. A systematic review of the literature demonstrated a wide variation in HV prevalence estimates due to several factors, such as HV diagnosis method, sex, age, study quality, and sampling method [2]. Previous investigated on self-recognized of HV, there was an error in rate of agreement with the diagnosis using radiologically assessed, and there is a problem that self-recognized cases is more likely to be perceived as less severe than the diagnosed cases by the physician [10]. Thus, a system that can correctly determine HV severity is desirable. Graded retests and inter-rater reliability using the Manchester scale (MS) were found to be excellent [11], and MS scale method can be widely applied in the future. The mean hallux abductus angle, measured using the radiographs of participants with an MS score of 2, whom this study was based on, was approximately 15°, which is the generally accepted minimum value for HV diagnosis [12]. HV can be influenced by external factors, e.g., wearing of high heels [13, 14] and injuries of the medial collateral ligament of the first metatarsophalangeal joint [15], and internal factors, e.g., genetic predisposition, sex, age, and flat feet [14]. Thus, HV is caused by a combination of factors, which indicates the importance to rank the relevant factors. However, to the best of our knowledge, studies investigating the factors related with HV using MS are scarce.

Support vector machine (SVM) is a machine learning-based classification method that can be applied to pattern recognition and regression analysis [16]. In the 1990s, the application of SVM was extended to nonlinear discriminant methods combined with kernel methods. SVM, which has been used to construct nonlinear discriminant functions by kernel tricks, is currently the best learning method for pattern recognition. In general, many features are used for pattern recognition and discrimination, and features are identified using feature selection methods, e.g., recursive feature elimination (RFE) [17].

SVM-RFE is one of the most widely used feature selection methods owing to its flexibility and simplicity. It can be applied to any model and produces the optimal set of features to achieve the best performance. In this study, we used SVM-RFE to analyze HV-related factors. Studies have applied this two-class classification algorithms using SVM and reported that the use of evaluation and diagnosis systems in the medical field has increased in recent years [18]. The RFE algorithm for nonlinear kernel allows ranking of variables but not comparison of the performance of all variables in a specific iteration.

Therefore, this study aimed to demonstrate the importance of HV-related factors via feature selection using SVM-RFE. Foot alignment was added to the basic items of age, sex, weight, and body mass index (BMI), and HV-related factors were analyzed via SVM-RFE.

## Methods

### Research design

This cross-sectional study included 928 participants aged ≥ 18 years. The participants were examined as a part of a foot health survey at sports and local health events, and a corporate health project was held in Osaka Prefecture in 2018–2020. The inclusion criteria were no pain or slight foot pain during loading and ability to walk. Among the participants, 48 for whom the date the photograph was taken was unclear and 16 for whom both feet could not be photographed were excluded. As a result, only 864 participants (353 males) were finally included in the analysis.

The collected data were analyzed to evaluate the accuracy of the extraction method for HV-related factors and feature selection via SVM-RFE using machine learning. The causal relationship between HV and the selected features was statistically investigated.

The study protocol was approved by the Research Ethics Review Committee of Osaka Kawasaki Rehabilitation University (approval no. OKRU29-A019). Furthermore, the study was conducted in accordance with the principles of the Declaration of Helsinki. The purpose and methods of the study were fully explained in advance to the participants, and measurements were performed after obtaining written informed consent. In addition, this study has been reported according to the STROBE guideline [19].

### Evaluation using the MS and foot measurements

The summed scores of the MS on the horizontal image of the forefoot body surface were used to evaluate the presence or absence of HV [8]. A digital camera (RX-0, SONY, Tokyo, Japan) was used to capture foot images for MS. A horizontal image of the forefoot was taken to confirm that the second toe was in the middle position of adduction and abduction as much as possible. All participants were evaluated for HV using the MS by one examiner, a physiotherapist with 21 years of general physiotherapy clinical experience.

**Fig. 1:**
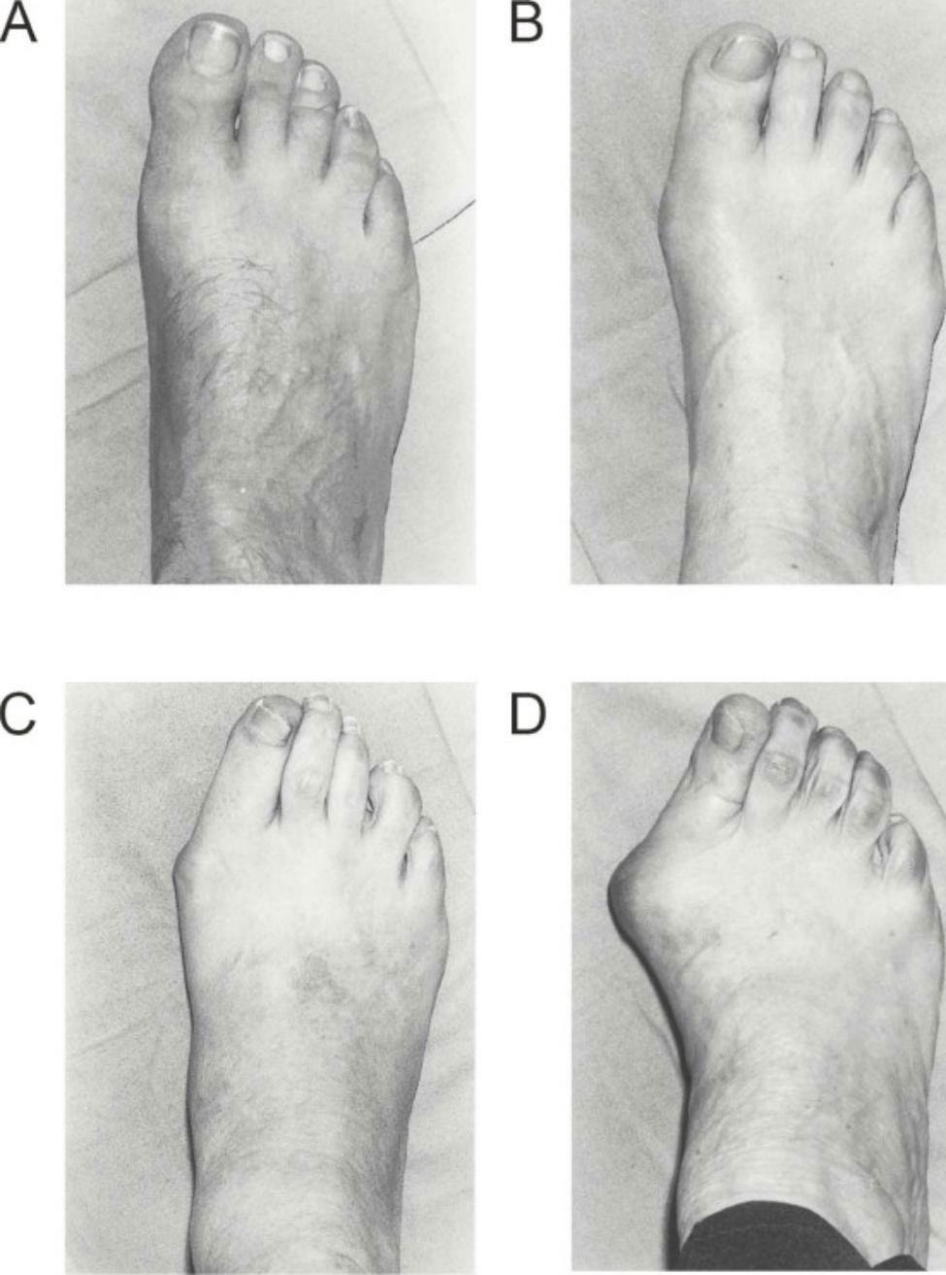
Evaluation of hallux valgus using the Manchester scale score. (A) No deformity (score = 0), (B) mild deformity (score = 1), (C) moderate deformity (score = 2), (D) severe deformity (score = 3). Diagram adapted from *Garrow et al* [8]

The criteria for the presence of HV were based on the grade classification of HV in MS: 0, no deformity; 1, mild deformity; 2, moderate deformity; and 3, severe deformity　(Fig. 1) [11]. If the summed score of the right and left MS was ≥ 4, the patient was considered as having HV. In this study, the standard value indicating the presence of HV was 2 points on both sides or ≥ 3 points on one side, and a total of ≥ 4 points.

Furthermore, dorsal height (DH), foot length (FL), and arch height ratio (AHR) were evaluated [20]. In this study, the DH/truncated FL (TFL) was defined as the AHR. DH, FL, and TFL were measured using a foot arch height-measuring instrument (Takei Corp, Niigata, Japan). The mean of both feet measurements was used for SVM-RFE analysis.

### Discriminant evaluation using SVM-RFE

SVM is a machine learning-based classification method that can be applied to pattern recognition and regression analysis. SVM-RFE was implemented using the RFE class in the feature selection module of scikit-learn. The SVM-RFE inputs were age, height, weight, BMI, average left–right FL, average DH, and average left–right AHR, all of which are numerical data. However, because the mean and scale of all items are different, the data were first standardized, and the mean and variance were set to 0 and 1, respectively. Then, we input the binary data of sex, history of foot injury, foot pain, and exercise habits and performed feature selection.

In this study, the linear function was used as the kernel function for feature selection. The value of the cost parameter (C), which determines tolerance for misclassification, needs to be determined and the accuracy of the prediction model needs to be evaluated. In the evaluation experiment, the fit rate, reproducibility, and accuracy of the model when C = 1.0 were obtained via a tenfold cross-validation.

### Statistical examination

In this study, the HV-related factors were extracted via feature selection using SVM-RFE, and their accuracy was evaluated. The SVM-RFE classification was implemented at the Tokushima University. The extracted factors were statistically processed, and normality of the explanatory variables was confirmed using the Shapiro–Wilk test. Comparison of the basic attributes between male and female participants was performed using the unpaired *t*-test or Mann–Whitney U test. The χ^2^ test or Fisher’s exact test was used for analyzing the sex-related difference in participants with and without HV. Pearson’s or Spearman’s correlation coefficients were used to examine the correlation between MS score and each explanatory variable in female participants with HV. Statistical analysis was conducted using IBM SPSS Statistics version 28.0 (IBM Corp., Armonk, NY, USA) with a significance level of 5%.

## Results

### Comparison of sex differences in basic attributes and foot measurements of the participants

Data were collected from 928 participants aged 18–96 years, including university students, workers, citizen athletes, and community-dwelling older people. Among them, 48 participants with unclear imaging data and 16 whose feet could not be photographed were excluded. As a result, data from 864 participants (353 men and 511 women) were used in the final analysis. The questionnaire was self-administered and contained the items age, sex, height, and weight.

Comparison of the basic attributes and foot measurements between the male and female participants is demonstrated in Table 1. The results indicated that the male participants had significantly higher height, weight, BMI, FL, DH, AHR, and total MS score than the female participants. Female participants were significantly older than male participants.

**Table 1:** Basic attributes and foot measurements of the subjects

	Male (n = 353)	Female (n = 561)	
	Mean ± SD	Mean ± SD	p-value
Age (y)	44.6 ± 21.2	56.7 ± 21.1	< 0.001
Height(cm)	169.9 ± 7.6	154.6 ± 6.4	< 0.001
Weight (kg)	66.5 ± 9.9	52.1 ± 8.0	< 0.001
BMI (kg/m^2^)	23.0 ± 3.0	21.7 ± 3.0	< 0.001
FL(mm)	247.6 ± 12.4	224.4 ± 4.6	< 0.001
DH (mm)	63.4 ± 5.2	56.0 ± 4.6	< 0.001
AHR (%)	25.6 ±2.2	25.0 ±2.1	< 0.001
MS Total Score	2.3 ± 1.8	1.4 ± 1.4	< 0.001

BMI, Body mass index; DH, Dorsal height; FL, Foot length; TFL, Truncated foot length; MS, Manchester scale

*: statistically significant, *P* < 0.05

### Predictive model accuracy evaluation of feature selection using SVM-RFE

The results of the tenfold cross-validation using SVM-RFE are presented in Table 2. The features selected by training the prediction model were age, sex, weight, and mean DH, with the number of features selected for the parameters being 10, 10, 9, and 1, respectively. Age, sex, and weight were mainly related to HV.

**Table 2:** Feature selection of hallux valgus-related factors using SVM-RFE

Selected features	Number of choices
Age	10
Sex	10
Weight	9
Dorsal height	1

SVM-RFE, support vector machine-recursive feature elimination

The accuracy evaluation results of the prediction model are presented in Table 3. The fit, repeatability, and accuracy of the model were determined via a tenfold cross-validation, with the results being 30%, 73%, and 43%, respectively.

**Table 3:** Accuracy evaluation using SVM-RFE

	Precision ratio	Recall	Accuracy
With HV	30%	73%	43%
Without HV	92%	64%	75%

HV, hallux valgus; SVM-RFE, support vector machine-recursive feature elimination

### Sex difference in the HV rate

Table 4 presents the HV rates by sex as an associated factor, with 24.9% of the female participants having HV compared with 7.6% of the male participants.

**Table 4:** Comparison of sex differences in hallux valgus

	Male	Female	p-value
	n (%)	n (%)	
With HV	27 (7.6)	127 (24.9)	p < 0.001
Without HV	326 (92.4)	384 (75.1)	
HV, hallux valgus			χ^2^test

### Comparison of the percentage of male and female participants with HV based on their age group

The proportion of the participants by age group is presented in Table 5. The age groups were classified based on the life stages (adolescence, adulthood, and old age) presented in Health Japan 21, a guideline issued by the Ministry of Health, Labor and Welfare.

**Table 5:** Comparison of the percentage of men and women with HV by age group

	Female				Male				
	With HV		Without HV		With HV		Without HV		
Age group	n (%)	Expected number	n (%)	Expected number	n (%)	Expected number	n (%)	Expected number	p-value
*18–29*	8 (9.1)	21.9	80 (90.9)	66.1	1 (0.8)	9.8	127 (99.2)	118.2	<0.01
*30–44*	10 (13.3)	18.6	65 (86.7)	56.4	0 (0)	3.8	50 (100)	46.2	<0.01
*45–64*	20 (23.3)	21.4	66 (76.7)	64.6	7 (7.4)	7.3	88 (92.6)	87.7	<0.01
*≥65*	89 (34.0)	65.1	173 (66.0)	196.9	19 (23.7)	6.1	61 (76.3)	73.9	0.08

The proportion of female participants with HV increased with increasing age, with 89 (34%) participants aged ≥ 65 years, followed by 20 (23.3%) aged 45–64 years, and 10 (13.3%) aged 30–44 years. Similarly, the proportion of male participants with HV increased with increasing age, with 19 (23.7%) participants aged ≥ 65 years, followed by 7 (7.4%) aged 45–64 years. The proportion of female participants aged ≥ 65 years was higher than that of male participants.

In the comparison of sex differences by age, significant differences were observed in the proportion of women with HV in the age groups of 18–29, 30–44, and 45–64 years but not in the age group of ≥ 65 years.

### Correlation between HV explanatory variables and total MS score

The correlations between HV explanatory variables and total MS score for female participants with a high prevalence of HV are presented in Table 6, and significant correlations were observed for age (r = 0.343), height (r = − 0.324), and FL (r = − 0.216) in female participants with HV in all age groups. In the significant correlation analysis were found for age (r = 0.478), height (r = − 0.483) in female participants with HV MS score in the age group of 45–64 years. No item showed a significant difference in female participants aged ≥ 65 years.

**Table 6:** Correlation between HV explanatory variables and MS score in females

Age group	Age (y)	Height (cm)	Weight (kg)	BMI (kg/m^2^)	FL (mm)	DH(mm)	AHR (%)
	r	r	r	r	r	r	r
*All age (n = 127)*	0.343*	−0.324*	−0.082	0.072	−0.216*	−0.019	0.080
*18–29 (n = 8)*	0.516	−0.126	−0.191	−0.126	−0.258	0.252	0.127
*30–44 (n = 10)*	−0.28	0.114	−0.309	−0.572	−0.076	0.038	0.076
*45–64 (n = 20)*	0.478*	−0.483*	−0.048	0.127	−0.142	−0.037	0.087
*≥65 (n = 89)*	−0.022	−0.153	−0.085	0.049	−0.085	−0.114	−0.072

BMI, Body mass index; FL, Foot length; DH, Dorsal height; AHR, Arch height ratio

Pearson’s or Spearman’s correlation. Statistically significant, * *P* < 0.05

## Discussion

To date, few studies have used machine learning to analyze joint deformities, and to the best of our knowledge, SVM-RFE has only been used for Kashin–Back disease, which involves alteration of hands [21]. In the present study, a feature selection algorithm was used to analyze HV-related factors using SVM-RFE.

The fit rate, reproducibility, and accuracy of the HV prediction model in this study were 30%, 73%, and 43%, respectively. This indicates that the explanatory variables of age, sex, weight, and DH were significantly related in 30% of the participants with HV but not in 73% of those without HV.

The final output of the SVM-RFE algorithm is a list of variables ranked according to their relevance. SVM-RFE is essentially a backward elimination method. However, the top-ranked variables are not necessarily the most relevant variables under the most relevant conditional on the specific ranked subset in the model [18]. Thus, the importance of HV-related factors depends on the number of variables. SVM-RFE algorithm allows the classification and ranking of variables but not the comparison of the performance of all variables, In other words, it is important to interpret the results in terms of their relationship to the response variable and other variables and the magnitude of the relationship. Therefore, in this study, statistical analyses were performed for HV-related factors.

Regarding feature-selected factors of HV, age, sex, weight, and DH were selected in that order. Statistical analysis of the selected factors revealed that women were predominant in terms of the sex ratio for HV. In this study, 24.9% of the female participants had HV compared with 7.6% of the male participants. In a questionnaire survey of 4,249 cases in the UK, HV was detected in 28.4% cases, with an odds ratio of 2.64, which was higher in men than in women [9]. In a study conducted on Japanese participants, the incidence rate of definite radiographic HV was 29.8%. Female sex was significantly associated with increased risk for HV (odds ratio, 1.71) [22]. A similar trend was observed in this study.

The mean age was higher in female participants with HV than in those without HV, and total MS score was found to be positively correlated with age (r = 0.478) in the age group of 45–64 years, suggesting that prime age is an important factor related to HV. Regarding age group, the proportion of HV was the highest in the older age group (aged ≥ 65 years) for both sexes but decreased in the younger age groups. In adolescents, HV was more common in female participants than in male participants, but sex difference was not observed in the age group of ≥ 65 years. As for the relationship between age and sex and HV onset, HV began to develop in female participants when they were in primary and secondary schools [2]. These findings are similar to the findings of the present study; however, in the present study, the incidence rate of HV was not different between older men and women. In an analytical study of 11,714 Japanese individuals aged 60–79 years, the incidence of HV increased with age in both men and women, with a significant increase observed between the age groups of 40–50 years and 50–60 years [23]. To prevent worsening of HV in women, it is important to inform them of appropriate shoe selection and preventive exercises for HV, especially for those aged ≥ 45 years.

The next most frequently extracted item in feature selection was weight, which did not show a valid statistical relationship with HV. In addition to weight, age, sex, obesity, inappropriate shoes, and physical activity were reported as risk factors for foot problems [13]. Studies have demonstrated that obesity is associated with reduced foot arches [24, 25]. Thus, obesity was reported to be associated with foot problems; however, in the present study, no correlation was observed between body weight and HV.

Foot height, which was extracted less frequently in feature selection, was not found to have the same trend as body weight in the statistical analysis. Foot height was related to arch deformities, such as flat feet and high arches, and previous studies have demonstrated that HV and flat feet are related [14]. However, some studies reported that HV was not related to flatfoot [26], and the correlation between foot arch reduction and HV development is still under debate. Furthermore, height was not correlated with HV in women aged 45–64 years, and it was not extracted in feature selection. This association between height reduction and HV may be due to fact that risk of spinal degeneration increases in HV (odds ratio1.75) [27], and not only the HV but also the alignment of other body parts tend to change in older age.

This study had some limitations. First, we used a cross-sectional design, and the causal relationship with HV onset cannot be fully explained. To conduct a more accurate analysis of HV-related factors, more specific models focusing on the severity of the deformity using a large dataset should be developed. Second, there were no data on the shoes used, such as high heels, which are considered to be related to HV, or on heredity. If these data were available, the accuracy and precision of the fit would further improve. Because HV is often treated with surgery when foot pain is severe and affects the patient’s quality of life, the factors associated with HV and foot pain should be examined in the future.

## Conclusion

The results of this study indicated that age, sex, and weight were the most frequently extracted features; however, age and sex showed significant differences in the subsequent statistical analysis. Although SVM-RFE is an effective method for feature selection, further analysis using a large dataset is needed to show the causal relationship between feature selection and weight.

## Data Availability

The dataset used and analyzed during the current study is available from the corresponding author on reasonable request.

## Abbreviations

SVM-RFE: Support Vector Machine-Recursive Feature Elimination
HV: Hallux Valgus
BMI: Body Mass Index
DH: Dorsal Height
FL: Foot Length
AHR: Arch Height Ratio
TFL: Truncated Foot Length
MS: Manchester Scale
